# *Helicobacter pylori *infection is not associated with an increased hemorrhagic risk in patients in the intensive care unit

**DOI:** 10.1186/cc4920

**Published:** 2006-05-16

**Authors:** René Robert, Valérie Gissot, Marc Pierrot, Leila Laksiri, Emmanuelle Mercier, Gwenael Prat, Daniel Villers, Jean-François Vincent, Michel Hira, Philippe Vignon, Patrick Charlot, Christophe Burucoa

**Affiliations:** 1 Réanimation Médicale, CHU Poitiers, 2 rue de la milèterie, BP 577 86021 Poitiers cedex France; 2 Réanimation Polyvalente, Hopital Girac 16140 Saint Michel France; 3 Réanimation Médicale, CHU Angers 4 rue Larrey 49100 Angers France; 4 Réanimation Chirurgicale, CHU Poitiers, 2 rue de la milèterie, BP 577, 86021 Poitiers cedex France; 5 Réanimation Médicale, CHU Bretonneau, 2 Boulevard Tonnelé 37044 Tours, France; 6 Réanimation Médicale, CHU de la Cavale Blanche rue Tanguy Pringent 29200 Brest, France; 7 Réanimation Médicale, CHU Nantes, 1 place Alexis Ricordeau 44093 Nantes cedex, France; 8 Réanimation Polyvalente, Centre hospitalier de Saintes, 9 place du 11 novembre BP 326, 17108 Saintes cedex, France; 9 Réanimation Polyvalente Chateauroux, Centre hospitalier de Chateauroux 216 avenue de verdun 36000 Chateauroux, France; 10 Réanimation Polyvalente Limoges, CHU Dupuytren 2 avenue Martin Luther King 87042 Limoges cedex, France; 11 Réanimation Polyvalente Niort, 40 avenue du général de Gaulle 79000 Niort, France; 12 Laboratoire de Microbiologie A EA 3807, CHU Poitiers, 2 rue de la milèterie, BP 577, 86021 Poitiers cedex France

## Abstract

**Introduction:**

The potential role of *Helicobacter pylori *in acute stress ulcer in patients in an intensive care unit (ICU) is controversial. The aim of this study was to determine the frequency of *H. pylori *infection in ICU patients by antigen detection on rectal swabs, and to analyze the potential relationship between the presence of *H. pylori *and the risk of digestive gastrointestinal bleeding.

**Methods:**

In this prospective, multicenter, epidemiological study, the inclusion criteria were as follows: patients admitted to the 12 participating ICU for at least two days, who were free of hemorrhagic shock and did not receive more than four units of red blood cells during the day before or the first 48 hours after admission to the ICU. Rectal swabs were obtained within the first 24 hours of admission to the ICU and were tested for *H. pylori *antigens with the ImmunoCard STAT! HpSA kit. The following events were analyzed according to *H. pylori *status: gastrointestinal bleeding, unexplained decline in hematocrit, and the number of red cell transfusions.

**Results:**

The study involved 1,776 patients. Forty-nine patients (2.8%) had clinical evidence of upper digestive bleeding. Esophagogastroduodenoscopy was performed in 7.6% of patients. Five hundred patients (28.2%) required blood transfusion. *H. pylori *antigen was detected in 6.3% of patients (95% confidence interval 5.2 to 7.5). *H. pylori *antigen positivity was associated with female sex (*p *< 0.05) and with a higher Simplified Acute Physiology Score II (SAPS II; *p *< 0.05). *H. pylori *antigen status was not associated with the use of fiber-optic gastroscopy, the need for red cell transfusions, or the number of red cell units infused.

**Conclusion:**

This large study reported a small percentage of *H. pylori *infection detected with rectal swab sampling in ICU patients and showed that the patients infected with *H. pylori *had no additional risk of gastrointestinal bleeding. Thus *H. pylori *does not seem to have a major role in the pathogenesis of acute stress ulcer in ICU patients.

## Introduction

*Helicobacter pylori *(*H. pylori*) is able to colonize gastric mucus and has a major pathogenic role in peptic ulcer [1], gastric cancer and MALT (mucosa-associated lymphoid tissue) lymphoma [2]. *H. pylori *is estimated to colonize more than 50% of the population worldwide [3,4], but its prevalence falls with improving living standards and hygiene [5].

The potential role of *H. pylori *in acute stress ulcer in patients in the intensive care unit (ICU) is more controversial. Indeed, the pathogenesis of stress ulcers has multiple causes, such as mucosal ischemia and/or ischemia-reperfusion, acid back-diffusion, and bile reflux [6,7]. Some of these factors have been linked to *H. pylori *infection [2,8]. Animal studies show that *Helicobacter *infection can contribute to the pathogenesis of acute stress ulceration [9,10]. Robertson and colleagues showed a tentative link between *H. pylori *seropositivity and the severity of gastric bleeding [11]. Similarly, Van der Voort and colleagues reported a significant correlation between *H. pylori *infection and the severity of upper gastrointestinal lesions in ICU patients [12]. Conversely, other studies have shown no relationship between *H. pylori *seropositivity and gastric bleeding [13,14].

*H. pylori *infection is difficult to detect in ICU patients. Direct isolation by ulcer biopsy is rarely possible because of the bleeding risk. Serologic testing is simple and has been used in several studies [11,13,14] but cannot discriminate current from past infection, and the antibody titer can be affected by hemodilution. *H. pylori *infection can also be detected by the [^13^C] urea breath test [15], but this technique cannot be used routinely, especially in ICU patients. *H. pylori *antigen detection in stool samples was recently validated with a 93.8% sensitivity and 96.0% specificity [16,17]; the method is noninvasive and a positive result is indicative of active infection. A rectal swab may appear to be an easy way to collect stool samples in ICU patients, because it is routinely done in ICU to detect colonization with multiresistant bacteria. Stool antigen testing on a rectal swab seems to be the more appropriate test for use on ICU patients, for whom techniques such as serology do not necessarily indicate an active infection, the urea breath test needs heavy technical adaptation to ventilated patients, and invasive methods are undesirable.

We used the stool antigen testing method on a rectal swab to study the prevalence of *H. pylori *infection in ICU patients, and to analyze the possible relationship of *H. pylori *infection to the risk of upper gastrointestinal bleeding.

## Materials and methods

### Patients and sampling

This multicenter prospective epidemiologic study was conducted in 12 ICUs (six in teaching hospitals and six in general hospitals). Patients were eligible for the study if they were admitted to a participating ICU for at least two days from January to August 2004. Patients were ineligible if their ICU stay was less than 48 hours, and patients with a previous history of gastric or duodenal ulcer were excluded from the study. Because the aim of the study was to analyze the potential role of *H. pylori *occurring during the ICU stay, patients who had hemorrhagic shock on admission or who received more than four units of red blood cell transfusion before or during the first 48 hours after admission to the ICU were also excluded from the study.

Rectal swabbing for *H. pylori *antigen detection was done within 24 hours after admission, at the same time as routine screening for multiresistant bacterial colonization in accordance with ICU policies. All swabs were kept frozen at -20°C. Stored samples were tested simultaneously for *H. pylori *antigen every 2 months. The results for *H. pylori *detection were not available until after the end of the study.

### Detection of *H. pylori *antigen

Preliminary tests with *H. pylori *antigen-positive stools allowed us to confirm good conservation of specific antigens on frozen rectal swabs. All rectal swabs were tested for *H. pylori *antigen with the ImmunoCard STAT! HpSA kit (Bioscience Europe, Nice, France) as recommended by the manufacturer. In brief, stored specimens were returned to room temperature just before testing. Each swab was placed in a tube containing 1 ml of sample diluent and was vortex-mixed for 15 seconds. The tip of the vial was snapped off, and four drops were added to the sample port of the test cassette. The test was read after incubation for exactly 5 minutes at ambient room temperature. Tests were recorded as positive if there was a blue line in the control window and a pink line in the test window, and negative if there was a blue line in the control window and no pink line in the test window.

### Serological study

Two of the 12 centers also tested some patients for *H. pylori*-specific IgG antibodies in serum, using the Platelia enzyme immunoassay (Bio-Rad, Marnes-la-Coquette, France), in accordance with the manufacturer's instructions. The test was positive if the ratio between the optical density of the specimen and the mean optical density of the control was 1 or more.

### Clinical data

The following clinical characteristics were recorded: age, gender, Simplified Acute Physiology Score II (SAPS II) on admission, reason for admission to the ICU, previous significant disease, intubation and mechanical ventilation, catecholamine infusion, and extra-renal procedures. The occurrence and origin of bleeding complications during the ICU stay were recorded. Upper gastrointestinal bleeding was suspected if the decline in hemoglobin concentration was associated with melena, or if there was an isolated unexpected decline in hemoglobin level higher than 2 g/dl within 48 hours or 1 g/dl on two consecutive days. The indication of esophagogastroduodenoscopy was left free to the physician in charge of the patient. The number of units of red blood cell transfused, whatever the evidence of upper gastrointestinal bleeding, was also recorded. Red blood cell transfusions were indicated by the physician in charge of the patients. For the purpose of the study there was no recommendation relating to the hemoglobin level; however, blood transfusions were usually prescribed in accordance with French consensus guidelines (hemoglobin level below 7 g/dl, or below 10 g/dl in an at-risk patient) [18].

The study was approved by the Ethics Committee of the French Society of Critical Care Medicine (Société de Réanimation de Langue Française).

### Statistical analysis

Data are expressed as means ± SD or as median and range, as appropriate. Qualitative values were compared by using the χ^2 ^test or Fisher's exact test, as appropriate. Continuous values were compared with Student's *t *test or analysis of variance for normal values, or with the Mann-Whitney test for nonparametric data. *p *< 0.05 was considered to denote statistical significance.

The factors associated with bleeding during ICU stay were also studied with multivariate analysis. Variables with *p *< 0.25 in univariate analysis were selected. A multivariate logistic regression model with bleeding event during ICU stay as the dependent variable was fitted in a forward stepwise procedure by using SAS (SAS Institute, Cary NC, USA). Predictive values are presented as odds ratios (ORs) and corresponding to 95% confidence intervals (CIs).

## Results

In the study, 2,266 patients were enrolled. Of these, 397 patients were excluded because their ICU stay lasted less than 48 hours or because they had previous history of gastric or duodenal ulcer. A further 93 patients were excluded because they required blood transfusion for bleeding on admission. Thus, 1,776 patients constituted the study group. The clinical characteristics of the patients are summarized in Table 1. The patients were admitted for medical reasons in 79% of cases, for emergency surgery in 13%, and for trauma in 8%. The main underlying diseases were alcoholism (21.8%), chronic obstructive pulmonary disease (18.5%), diabetes mellitus (16.6%), cancer (14.6%), chronic heart failure (13.0%), chronic renal failure (5.9%), hematologic malignancies (4.7%), and cirrhosis (3.7%).

On the day of admission to the ICU, 10.3% and 2.6% of the patients, respectively, were receiving aspirin and anti-inflammatory agents, 6.5% were receiving corticosteroids and 7.8% were on anticoagulation therapy; 27.9% were receiving antimicrobial therapy for a mean duration of 5.7 days (range 1 to 27) and 15.8% were receiving anti-ulcer prophylaxis (usually proton pump inhibitors).

*H. pylori *antigen was detected in 6.3% of patients (95% CI 5.2 to 7.5). *H. pylori *antigen positivity was associated with female sex (*p *< 0.05) and a higher Simplified Acute Physiology Score II (SAPS II) (*p *< 0.05). The other clinical characteristics did not differ according to *H. pylori *status (Table 2). The percentages of patients requiring red blood cell transfusions and the total numbers of units of blood cells transfused were similar in the two groups (Table 2).

During their ICU stay, 307 (17.3%) patients had clinical evidence of bleeding. Among these, 84 patients (27%) had extra-digestive bleeding, 156 (51%) had an unexplained decline in the hemoglobin level and 67 (22%) patients had clinical evidence of upper digestive bleeding. Esophagogastroduodenoscopy was performed in 7.6% of cases, for suspected gastrointestinal bleeding or an unexplained decline in the hemoglobin level, showing gastric or duodenal ulcer in 45%, oesophageal ulcer in 24% and diffuse gastritis in 12% of the cases. *H. pylori *antigen was positive in 2.5% of the patients with abnormal esophagogastroduodenoscopy. Five hundred patients (28.2%) required blood transfusion and 55 (3.1%) received more than four units of red blood cell transfusion. The mean number of units of red blood cells per transfused patient was 5.5 (range 1 to 69) for these latter patients. The characteristics of the patients with clinical evidence of bleeding (excluding those with extra-digestive bleeding) were compared with those of the patients without bleeding (Table 3). Survival was significantly better in patients without bleeding than in patients with clinical evidence of bleeding during their ICU stay (*p *< 0.001). Using multivariate analysis, the bleeding risk was independently associated with SAPS II (OR = 1.013, 95% CI 1.003 to 1.022), shock on admission (OR = 1.658, 95% CI 1.197 to 2.295), and creatinine plasma level on admission (OR = 1.001; 95% CI 1.00001 to 1.0013).

### Serology

Tests for *H. pylori*-specific antibodies were performed on admission in 312 patients in 2 of the 12 centers. The results were negative in 208 patients (67%) and positive in 104 patients (33%). Serology was concordant with antigen detection results in 66% of cases. The hematocrit fell in similar proportions of seropositive and seronegative patients (13.5% and 17.9%, respectively). The incidence of digestive gastrointestinal hemorrhage was 2.9% and 1.0% in seronegative and seropositive patients, respectively. The numbers of patients requiring blood transfusion were similar in the seronegative and seropositive groups.

*H. pylori *serology was compared with *H. pylori *antigen swab detection in the 312 patients. Antigen detection was negative in 91 seropositive patients and positive in 16 seronegative patients.

## Discussion

We found no correlation between *H. pylori *infection diagnosed by antigen detection on rectal swabs and the occurrence of hematocrit decrease or gastrointestinal hemorrhage during the ICU stay.

The prevalence of *H. pylori *based on stool antigen detection was only 6.3%. This percentage is much lower than that previously reported in industrialized countries and particularly in ICU patients (about 60%) [11,13]. This latter prevalence was significantly higher than the 39% reported in blood donor control population [11]. Several factors might explain the low prevalence of *H. pylori *infection found here by rectal swabbing. The sensitivity of this method may be influenced by a variable amount of stools recovered by swabbing. Furthermore, the *H. pylori *antigen detection method was validated on direct stool samples rather than swabs [16,17]. However, stool sampling can be difficult on admission in ICU patients because of intestinal ileus or impaired transit. The urea breath test, which is the reference method, had been performed in a restricted population that could not indicate a true prevalence [12], and this method cannot be used routinely in ICU patients. Because serological methods cannot discriminate recent from past infection, they may overestimate the frequency of *H. pylori *infection.

It is important to underline that, in our study, the seropositivity rate in a subgroup of 312 patients was 33%. This rate was significantly lower than in previous serological studies [5,14,19] and might corroborate the low rate found with the swab sampling. The exclusion from the study of the patients with a history of ulcers might have also contributed to this relatively low prevalence of *H. pylori *positivity in our population. Additionally, 27.9% of our patients were receiving antibiotics on admission to the ICU; these might have participated in the eradication of *H. pylori *[20]. According with our results, some studies suggest that the prevalence of *H. pylori *infection has been overestimated [21], and a recent investigation showed that the prevalence of peptic ulcer disease fell during a 10-year study period [22]. New epidemiological studies on *H. pylori *would be of interest to confirm this trend.

The frequency of patients who required red blood cell transfusion was 28.2% in our study, a rate close to that reported by Hebert and colleagues (25%) [23] but lower than that observed in a recent European survey (37%) [24]. However, it should be noted that we excluded patients who required significant red cell transfusions at about the time of admission to the ICU. The indications for red blood cell transfusion may vary with the type of ICU: Groeger and colleagues reported rates of 16% in a medical ICU and 27% in a surgical ICU [25]. The hemoglobin cutoff at which the ICU practitioners prescribed red blood cell transfusion was not recorded in our study, but they took account of the TRICC (Transfusion Requirement In Critical Care) study supporting a restrictive transfusion policy [26]. Similarly, contemporary French consensus guidelines recommended transfusion when the hemoglobin level fell below 7 g/dl, except in patients with ischemic myocardial disease, sepsis, or heart failure [18].

The incidence of digestive bleeding in our study (2.8%) was similar to the estimated prevalence in previous surveys [13,27]. In addition, 7.5% of our patients had an unexplained decrease in the hematocrit warranting gastroscopic examination. However, 28.2% of the patients required blood transfusion, indicating clearly that some patients were transfused without the information on upper gastrointestinal bleeding.

Robertson and colleagues observed a trend towards a significant relationship between *H. pylori *seropositivity and gastric bleeding in a series including 100 ICU patients [11]. Similarly, Van der Voort and colleagues found a significant correlation between [^13^C] urea breath test positivity and the endoscopic severity of upper gastrointestinal mucosal lesions in 44 ICU patients [12]. In a recent case-control study, Maury and colleagues showed that *H. pylori *infection, whatever method was used to detect it (serology, stool antigen detection or histologic examination), was more frequent in patients with upper gastrointestinal bleeding [28]. In contrast, no such relation was found with *H. pylori *seropositivity [13,14,19]. In two studies involving, respectively, 229 and 301 patients in cardiosurgical intensive care units, no link was found between *H. pylori *serostatus and upper gastrointestinal bleeding [14,19]. The seroprevalence in these studies was about 60% [14,18,19]. Our study of a very large number of ICU patients showed no relationship between fecal *H. pylori *antigen status and gastrointestinal bleeding. Indeed, the 111 patients with *H. pylori *antigen positivity, indicating a current proven *H. pylori *infection, did not have a higher incidence of gastrointestinal bleeding or higher transfusion requirements. Similarly, the subgroup of 104 seropositive patients was not associated with higher gastrointestinal bleeding or red blood cell transfusions.

## Conclusion

This large study showed a low prevalence of *H. pylori *infection in ICU patients, as diagnosed by antigen detection on rectal swabs. The patients infected by *H. pylori *were not at increased risk of gastrointestinal bleeding, suggesting that *H. pylori *does not have a major role in the pathogenesis of acute stress ulcer in ICU patients.

## Key messages

Antigen detection of H. pylori on rectal swab was positive in 6.3% of the ICU patients.

The patients infected with *H. pylori *had no additional risk of gastrointestinal bleeding.

## Abbreviations

CI = confidence interval; ICU = intensive care unit; OR = odds ratio.

## Competing interests

The authors declare that they have no competing interests.

## Authors' contributions

RR and CB designed the protocol. RR, VG, MP, LL, EM, GP, JFV, MH, PV and PC were responsible for the inclusion of the patients and data collection. LL and CB conducted the microbiologic assay and serological study. RR, VG, MP and CB performed the data and statistical analysis. RR, VG, DV, PV and CB prepared the manuscript. All authors read and approved the final manuscript.

## Figures and Tables

**Table 1:** Clinical characteristics of the 1,776 ICU patients

Characteristic	Value
Age in years, mean ± SD (range)	61.0 ± 17.3 (15–100)
Male sex (%)	64
SAPS II	40.1 ± 16.4
Mac Cabe 1 (%)	49.8
Mac Cabe 2 (%)	41.0
Mac Cabe 3 (%)	9.2
Mechanical ventilation (%)	77.0
Mechanical ventilation duration (days)	10.4 ± 14.6
Shock on admission (%)	23.5
Sepsis (%)	21.4
Creatinine level on admission (μmol/L)	145 ± 185
Extra-renal therapy (%)	11.5
ICU stay (days)	14.6 ± 16.0
ICU mortality rate (%)	20.2

SAPS, Simplified Acute Physiology Score.

**Table 2:** Clinical characteristics of ICU patients with negative and positive *H. pylori *antigen detection

Characteristic	Hp^- ^(*n *= 1,665)	Hp^+ ^(*n *= 111)
Age (years)	60.8 ± 17.3	63.6 ± 15.8
Male/female (%)	65/35	55/45*
SAPS II	39.9 ± 16.5	43.3 ± 15.6*
Patients with mechanical ventilation (%)	77.0	77.5
Duration of mechanical ventilation (days)	12.1 ± 14.0	12.3 ± 12.1
Patients with sepsis (%)	21.3	22.7
Patients receiving antibiotics on admission (%)	27.8	29.7
Creatinine on admission (μmol/l)	144 ± 185	156 ± 182
Ulcer prophylaxis (%)	15.9	11.7
Hemoglobin on admission (g/dl)	12.4 ± 7.8	11.5 ± 2.6
Patients with hematocrit fall (%)	13.6	12.6
Patients requiring red blood transfusion (%)	28.0	29.7
Red blood transfusion in patients requiring transfusion (units)		
Mean ± SD	5.5 ± 6.7	3.9 ± 3.8
Median (range)	3 (1–69)	3 (1–43)
Death (%)	23.4	20.0

Hp^-^, negative for *H. pylori *antigen; Hp^+^, positive for *H. pylori *antigen; SAPS, Simplified Acute Physiology Score. * *p *< 0.05.

**Table 3:** Main clinical characteristics of patients with and without bleeding during their ICU stay

Characteristic	Bleeding patients (*n *= 223)	Non-bleeding patients (*n *= 1,469)
Age (year)	61.2	61.0
Male sex (%)	68.2	63.4
SAPS II	44.7 ± 17.8	39.2 ± 15.9^a^
Patients with mechanical ventilation (%)	78	77
Patients with sepsis (%)	27.4	20.3^b^
Shock on admission (%)	35.9	21.2^a^
Creatinine on admission (μmol/l)	180 ± 176	135 ± 175^b^
Positivity of *H. pylori *antigen (%)	5.4	6.3
Death (%)	30.9	18.6^a^

The patients with documented extra-digestive bleeding were excluded from this analysis.

^a^Statistical significance: *p *< 0.01 with univariate analysis. ^b^Statistical significance: *p *< 0.05 with univariate analysis.
